# Advance Care Planning in Multicultural Communities: A Document Analysis of Resources to Support Healthcare Staff and Consumers in Australia

**DOI:** 10.1111/hex.70460

**Published:** 2025-10-15

**Authors:** Upma Chitkara, Ashfaq Chauhan, Ramya Walsan, Mary Li, Maha Pervaz Iqbal, Nadine El‐Kabbout, Misbah Faiz, Vitor M. Rocha, Ursula M. Sansom‐Daly, Reema Harrison

**Affiliations:** ^1^ Centre for Health Systems and Safety Research, Australian Institute of Health Innovation Macquarie University North Ryde Sydney Australia; ^2^ Campbelltown Hospital NSW Health Campbelltown New South Wales Australia; ^3^ Nafs Counselling Sydney New South Wales Australia; ^4^ Health Professionals Councils Authority, NSW Health Sydney New South Wales Australia; ^5^ Murray Primary Health Network Bendigo Victoria Australia; ^6^ Behavioural Sciences Unit, School of Clinical Medicine Discipline of Paediatrics and Child Health, UNSW Medicine & Health, UNSW Randwick Sydney Australia; ^7^ Kids Cancer Centre, Sydney Children's Hospital Randwick Sydney Australia; ^8^ Sydney Youth Cancer Service, Prince of Wales Hospital Nelune Comprehensive Cancer Centre Randwick Sydney Australia

**Keywords:** advance care planning, consumer engagement, culturally and linguistically diverse

## Abstract

**Introduction:**

Advance care planning (ACP) provides a person‐centric approach for discussing future care wishes that is responsive to individual preferences and needs. People from culturally and linguistically diverse (CALD) backgrounds have substantially lower opportunities for engagement in ACP, contributing to less person‐centred care, particularly towards the end of life. This study aimed to identify and describe the currently available resources in Australia that support healthcare staff and people from CALD backgrounds in engaging with ACP.

**Methods:**

Altheide's document analysis approach was used to systematically search and select eligible publicly available resources published between January 2013 and June 2023 from websites of government health departments and registered non‐government organisations that focused on facilitating ACP with people from CALD backgrounds. A narrative synthesis was performed to report the characteristics and scope of resources. Thematic analysis was employed with the content to identify considerations and recommendations for engaging with individuals from CALD backgrounds in ACP.

**Results:**

A total of 30 eligible resources were identified, of which 21 resources (70%) originated from government sources. Most of the resources targeted consumers (21/30, 70%), and a total of 91 community languages were covered across 15 resources that were offered in translated versions. Five resources were available in easy‐to‐read English. Thematic analysis of the ten resources that included considerations or recommendations for CALD populations identified four themes: practising culturally sensitive and person‐centred approach, supporting communication needs, staff recruitment and training, and provision of collaborative and multidisciplinary support.

**Conclusions:**

Whilst a large number of resources have been developed to facilitate uptake of ACP among the general population, a limited number of resources provide specific recommendations or support for working with CALD communities. Few resources reported the use of co‐design with communities despite recommendations to do so. Targeted information both for consumers and care providers is required to provide culturally relevant and inclusive support that promotes engagement in ACP towards person‐centric care.

**Patient and Public Involvement:**

The iCanCare Project described includes consumer investigators who have contributed to conceptualisation and design of the project, in addition to a Project Steering Group including consumer members who have guided the research process.

## 1 Introduction

Advance care planning (ACP) is a medical term which has been described as ‘the ability to enable individuals to define goals and preferences for future medical treatment and care, to discuss these goals and preferences with family and health‐care providers, and to record and review these preferences if appropriate’ [1]. ACP is relevant to consumers across the entire continuum of healthcare, including palliative and end‐of‐life care; it is the process of supporting individuals to explore and share their personal values, life goals and preferences regarding future healthcare, irrespective of their current age and health status [1, 2, 3]. In taking a person‐centred approach, ACP facilitates informed decision‐making by both families and healthcare staff and development of care plans towards patients' values, goals and personal preferences across the continuum of care [1, 2]. In Australia, ACP is identified as a component of high‐quality healthcare delivery and endorsed by the Australian government through its recognition in national care quality standards [4]. As a mechanism to plan for future care, ACP is particularly advised for individuals with a poor prognosis to exercise their autonomy in decision‐making for stages of life in which they are not capable of making decisions for themselves [5].

ACP can take many forms; an Advance Care Directive (ACD) is a formal, legally enforceable document recording a person's wishes and preferences about their future care and is more often used by patients. Like other settings internationally, ACP often occurs informally in Australia using a range of documents and through a variety of practices. ACP encompasses any instance of information sharing between healthcare staff, patients and/or their support person about their wishes, values and preferences for care [6, 7, 8]. As a result of enhanced person‐centred care and decision‐making, ACP has been associated with decreased hospitalisation, intensive care admission and invasive medical procedures towards the end of life [6, 7, 9, 10, 11, 12]. ACP has also been associated with reduced stress, anxiety and depression amongst patients and carers [6, 7, 9, 10, 11, 12].

Culture, ethnicity and language proficiency have been identified as factors contributing to people from CALD backgrounds having fewer opportunities to engage with ACP, leading to lower ACP uptake when compared to the general population [13]. Culturally and linguistically diverse (CALD) is a term used predominantly in Australia to describe people who were born overseas, speak languages other than the official national languages and/or have lower proficiency in native or national languages, and/or who have parents who were born overseas [6]. Australia has a large proportion of people from CALD backgrounds. One large prospective audit study of 4187 records of adults aged 65 years and over accessing primary care, hospital and long‐term care facilities demonstrated a lower prevalence of completed ACD among those born outside of Australia (21.9%) compared to those born in Australia (28.9%) [8]. Similarly, lower rates of documented ACP communication have been identified among people from CALD backgrounds compared with the general population [14]. With fewer opportunities to engage in ACP, people from CALD backgrounds may be more likely to be exposed to care that is not aligned with their preferences, which may include a higher risk of unwanted and burdensome health interventions towards the end of their life [15, 16].

Factors associated with lower uptake of ACP with CALD communities include low clinician confidence to deliver individualised and culturally sensitive conversations around ACP and a lack of awareness and understanding of ACP among people from CALD backgrounds [14]. ACP, being a Western concept, has also been argued as one of the reasons for its low uptake among CALD communities [17]. Differences in Western principles of individual autonomy versus Eastern collectivist culture of family‐oriented decision‐making were expressed as potentially impacting engagement with ACP [18]. Patient‐level characteristics, including years spent as a migrant in the country of residence, acculturation and cultural and religious beliefs, and the patient's desire to talk and plan about death, have also been associated with ACP uptake [13]. In light of these factors, a vast range of resources have been produced to provide information and approaches to encourage ACP uptake among CALD communities in the Australian population [19, 20, 21, 22, 23]. Previous studies have examined ACD templates and resources to support substitute decision‐makers and identified the extent to which religious and cultural information is captured in ACP documentation [24, 25]. To date, there is a lack of synthesis of the currently available guidance to facilitate the process of ACP among CALD communities. This study was conducted to address this evidence gap.

The study aimed to describe the currently available resources that support healthcare staff and health consumers from CALD backgrounds in Australia to engage with ACP and the proposed engagement practices within as part of a wider programme of research to explore ACP uptake among people from CALD backgrounds [26]. In the Australian healthcare context, consumers are people who use, have used or are potential users of health services, and this term has been identified by consumer organisations as the preferred term for this group [27].

## 2 Methods

Altheide's document analysis methodology was used to systematically search, select and analyse resources relevant to the research aim [28, 29]. This is a systematic document analysis method comprised of four steps: (1) document search, (2) document selection, (3) document appraisal and (4) data synthesis by organising into major themes and sub‐themes. This approach was used because of its prior application to explore a range of information resources within the health sector [30, 31], its ability to study varied forms of documents [29], and for the provision of evidence that was combined with qualitative interviews to minimise bias, establish credibility and guide the next steps as a part of a larger study [28]. The Standard for Reporting Qualitative Research guideline was used to report the research process (Supplementary File 1) [32].

### 2.1 Document Search

An iterative search and selection process was undertaken. A preliminary scoping search was conducted between October 2022 and March 2023 by three members of the research team (A.C., M.I. and M.L.) using key search terms (Table 1). These search terms were developed based on prior studies and refined based on the expert opinion of the Project Steering Group members who provided external oversight of the project [25, 33].

**Table 1:** Key search words.

Palliative care	Advance care	End‐of‐life care	Shared decision‐making
Palliative care planning Life support care Palliative care directive	Advance care directive Advance care planning Advanced personal plan	Life wishes Life support care	Communication Substitute decision‐maker Carer decision‐maker

The preliminary scoping search identified 86 resources comprising strategic plans, policy documents, frameworks, implementation plans, question guides, how‐to guides, fact sheets, toolkits, techniques for communication, education and training resources, ACD templates, medical procedures and treatment decision templates. The content of these 86 documents was used in consultation with the project lead (R.H.) to further refine the search strategy and develop eligibility criteria.

### 2.2 Eligibility Criteria

*Data Sources*: As primary contributors to the production of health planning resource, websites of Australian federal, state, territory and local level health departments, services and agencies were searched. Websites of registered non‐governmental organisations (NGOs) that provide support and resources to consumers and healthcare staff for ACP were also included.

*Document Type:* Media (printed, audio and video) that were available for use by healthcare staff and/or consumers that provide support, prepare and guide them for ACP were included. Resources published between January 2013 and June 2023 were eligible to focus on contemporary resources. Archived, redundant or outdated versions of media published during this time frame were excluded, as they were not considered reflective of sources that support current practice. Resources that provided guidance for either consumers or healthcare staff about planning or enacting ACP, such as how to organise thoughts and how to communicate about ACP (e.g., question guides, how‐to guides, fact sheets, toolkits, techniques for communication or other similar resources) specific to CALD populations were eligible.

#### 2.2.1 Exclusion Criteria

Documents that did not meet the above criteria were excluded. Strategic plans, policy documents and frameworks were excluded as they did not provide information relevant to the research aim. Implementation plans were excluded as they do not form a component of the planning process, but rather provide guidance on how to bring the plan to action. Education and training resources designed to provide education/upskilling were included, but those related to obtaining a qualification or professional development were excluded as they were considered too broad and warrant their own line of study. Media used to document ACP such as templates for ACDs, medical procedures and treatment decision templates were also excluded as they were not relevant to the research aim of resources to facilitate uptake of ACP as a process rather than the recording of decisions. Templates were considered as documents to record the outcomes of the communication process rather than those facilitating the process to occur. Resources that did not provide consideration or recommendations for CALD communities or staff working with CALD communities were not eligible.

*Unit of Analysis:* The key unit of analysis was information or guidance to inform how to communicate and/or how to prepare for ACP communication with individuals from CALD backgrounds. A purpose‐built data‐collection tool was developed to extract relevant information (Supplementary File 3). One researcher (U.C.) applied the eligibility criteria to fifteen websites in July 2023 (Supplementary File 2). The resulting documents were assessed against the eligibility criteria by one researcher (U.C.). The final group of documents was reviewed and agreed upon by the author team.

### 2.3 Data Extraction

Data extraction was conducted by two authors (M.L. and U.C.) in an iterative manner. Preliminary data extraction was conducted by one researcher (M.L.), where hyperlinks to identified resources were tabulated using MS Excel. Some hyperlinks led to a single resource, while some led to a single webpage containing multiple links to further documents. This process was followed by a thorough review of documents accessed via each hyperlink by a second reviewer (U.C.) to extract relevant resources. Data were then extracted from each eligible resource into the purpose‐built data extraction table using MS Word by the lead researcher (U.C.) (Supplementary File 3).

### 2.4 Data Synthesis and Reporting

Due to the large number of eligible documents, initial analysis included documents which were either translated into community languages and/or with specific considerations or recommendations for CALD groups. Final analysis included only those documents with specific considerations or recommendations for CALD groups. Thematic analysis was undertaken by three reviewers (U.C., R.W. and A.C.) using an inductive approach to identify the nature of resources available and the approaches that were identified as facilitating ACP with CALD communities [34]. CALD‐related information from eligible documents was tabulated on an Excel sheet, concepts of approaches for engagement around ACP were categorised and themes generated.

## 3 Results

### 3.1 Characteristics

**Figure 1:**
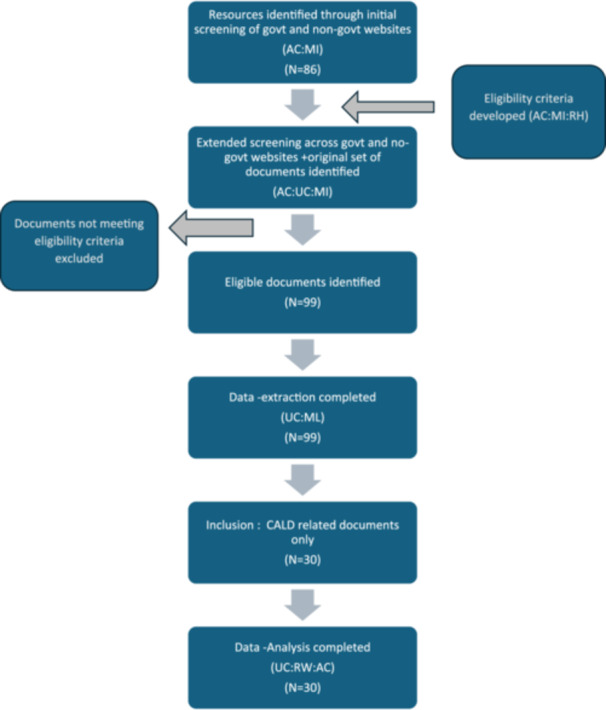
Document flow and selection.

A total of 30 unique resources were included in the analysis drawn from 17 sources (Figure 1). Of the 30 resources, many (*n* = 21) originated from government organisations (see Table 2) and the remainder from non‐government organisations. Most resources were at the direct‐care level (*n* = 28).

**Table 2:** Data extraction table.

Govt./Non‐govt.	Organisation, year	Level	Target group	Media type and aim	Key information	Available in language/s other than English	Recommendations for CALD community	Evidence base	Consumer involvement
Govt.	Advance Care Planning Australia, 2021 [19]	Federal	Consumers	Type: electronic (website) Aim: facilitate decision‐making	Create your plan. Online portal providing information on process to create ACP in each state of Australia and access to relevant forms	Yes 19 community languages: Arabic; Cantonese; Simplified Chinese; Traditional Chinese Croatian; Greek; Hindi; Italian; Japanese; Macedonian; Mandarin; Polish; Portuguese; Russian; Serbian; Spanish; Tagalog; Turkish; Vietnamese	No	No	No
Govt.	Australian Commission on Safety and Quality in Health Care, 2021 [20]	Federal	Healthcare staff	Type: print Aim: facilitate thinking about EOLC, facilitate communication, facilitate decision‐making	A user guide for delivering and supporting comprehensive EOLC (healthcare professionals) *A.‘Patient‐centred communication and shared decision making’ recognised as First element* *a. Starting the conversation:* b. Sharing decisions about care c. Tips for having a conversation about EOL *C. PREPARED Model—A guide for clinicians for conversations about the last days of life—provides a list of useful phrases, questions and ideas for each component* **P**repare for the discussion **R**elate to the person **E**xplore priorities and concerns **P**rovide information **A**cknowledge emotions and concerns **R** Foster Realistic hope **E**ncourage questions **D**ocument	No	*1. Tips for having a conversation about end of life*: Ask patients what matters to them. Patients will have different expectations, cultural and spiritual needs, and other personal preferences that may be important to their experience of death and dying (p. 9). *2. Considering spiritual and cultural needs:* Patients may choose to discuss goals with people that provide different types of support. Appreciating and allowing for cultural differences and diversity will improve the end‐of‐life experience for patients, families, carers and other support people. Spiritual care practitioners can help other members of the healthcare team understand the patient's spiritual needs (p. 13). *3. Response to concerns*: Allied health professionals are often critical in addressing concerns about mental, psychological, spiritual and cultural health (p. 18). *4. PREPARED Model—*A guide for clinicians for conversations about the last days of life—provides a list of useful phrases, questions and ideas for each component (Appendix A) **P**repare for the discussion **R**elate to the person: Consider cultural and contextual factors which may influence preferences **E**xplore priorities and concerns: Do you (or person's name) have any spiritual beliefs or cultural practices that we need to know about when thinking about the best care for you (or him/her)? Are there any special rituals that you would like to arrange after (person's name) dies? **P**rovide information **A**cknowledge emotions and concerns: Sometimes silence can be helpful, consider touch when culturally appropriate **R** Foster Realistic hope **E**ncourage questions: Check understanding and if information provided meets needs **D**ocument	Yes	No
Govt.	Cancer Australia, Govt. of Australia [21]	Federal	Consumers	Type: Print Aim: facilitate thinking about EOLC, facilitate communication, facilitate decision‐making	‘Finding the Words’ (booklet) Assists consumers to initiate conversations around EOL and talk with the people who can support them (family/friends), and their healthcare team.	Yes 8 community languages: Arabic; Cantonese; Croatian. Greek; Italian; Mandarin; Spanish; Ukrainian	No	No	Yes Pilot study with consumers post‐preparation
Govt.	Department of Health and Age Care, 2019 [22]	Federal	Consumers	Type: electronic (webpage) Aim: facilitate thinking about EOLC, facilitate communication, facilitate decision‐making	Start the Conversation Tips on starting conversation about dying/care preferences with further links to resources *Step 1. Understanding difference between palliative care and EOLC is a good place to start*. *Step 2. Thinking about:* *a. Palliative Care:* where would you like to be when having palliative care? How to get palliative care? Who would you like to provide the care? what kinds of treatment can you or can't you live with? *b. End‐of‐life care options:* what do you want for your end‐of‐life care? would your loved ones know what you want for your end‐of‐life care? who would make decisions about your end‐of‐life care if you couldn't? where would you like your care to be (home/hospital/hospice)? who would you want with you when you die? *Step 3. Start the conversation with your loved ones*.	Yes Six community languages: Arabic; Greek Italian; Simplified Chinese; Traditional Chinese; Vietnamese	No	No	No
Non‐govt.	End of Life Directions for Aged Care (ELDAC), 2022 [23]	Federal	Healthcare staff	Type: print Aim: facilitate thinking about EOLC, facilitate communication, facilitate decision‐making	XX	No	Supporting Inclusive End‐of‐Life Care: People from Culturally and Linguistically Diverse backgrounds Each section has actions for age care provides to support inclusive EOLC. *Making informed choices:* Provide information in an appropriate format, through different forms (online/hardcopy/newsletter/verbal) and in a language the consumer understands) *Adopting systemic approaches to planning and implementation:* Engage consumers in a culturally safe, supportive environment that enables them to participate as active partners, as well as articulate their individual needs. *Meeting the needs of the most vulnerable* Provide inclusive service models to address the needs of the most vulnerable, and work with other stakeholders to ensure that the full spectrum of needs is met.	Yes	No
Govt.	NSW health, 2021 [35]	State	Healthcare Staff: medical specialists, GPs, nurses, allied health	Type: print Aim: facilitate communication, facilitate decision‐making	End‐of‐Life Care and Decision‐Making: Guideline General Guidelines on developing a management plan for EOLC. • Process of EOL decision‐making • Assessment • Disclosure • Documenting discussions • Documenting consensus decision	No	1. Everyone receiving healthcare has a right to be informed about their condition/care options in the form of good quality health information, in a culturally responsive format and language that they can understand. (designed for CALD audiences, ETR versions, pictures) 2. Preference for autonomous decision‐making versus a collective or delegated approach to EOL decisions should be explored on an individual basis by engaging appropriate trained cultural health workers/interpreters 3. Avoid informal use of untrained interpreters while communicating essential clinical information 4. Culturally appropriate and responsive care at EOL is: (a) Supportive of personal, family and community needs (may include Palliative care at home, return to community and cultural or ritual activities) (b) Use of Multicultural health workers to work with the patient/family to inform service providers about specific needs.	Yes	Yes Guideline is based on a framework which was developed in consultation with consumer groups.
Govt.	NSW Health, 2021 [36]	State	Consumers	Type: print Aim: facilitate communication	Asking questions can help—For people when approaching the last Days of Life Questions to help consumers get the information they want about their illness and treatment options in the last days of life.	No	Questions include: 1. Is there anyone I can speak to about my spiritual/religious needs?2. Can I access an interpreter to speak to my family? 3. Space to note down phone contacts including Interpreter Service	No	Yes Collaboration with consumer advisors
Govt.	NSW Health, 2020 [37, 38, 39, 40, 41]	State	1–4 Consumers; 5 healthcare staff	Type: print Aim: facilitate thinking about EOLC, facilitate communication, facilitate decision‐making	XX	No	1. Palliative Care for you (Easy to Read) [31]: what is palliative care, where to get palliative care, how to make choices about palliative care. 2. Palliative care for someone close to you (Easy to read) [32]: what is palliative care, support available for someone who is dying, what to expect with palliative care. 3. Palliative Care for children (Easy to read) [33]: what is palliative care for children, where palliative care for children happens. 4. Talking about death and dying (Easy to read) [34]: What is death and how to talk about it 5. Death, dying and palliative care (HCPs) [35]: guide to how to use above 4 easy read resources. Easy Read materials [1, 2, 3, 4] are targeted to people with intellectual disability but are also useful resources for people with issues with low literacy or **with English as a second language**.	No (all)	1. Yes 2. Yes 3. Yes 4. Yes All 4 consulted consumers during the preparation phase and conducted a pilot study with consumers post‐preparation 5. No
Non‐govt.	Palliative Care, Victoria [42]	State	Consumers	Type: electronic (website) with videos and podcasts Aim: facilitate thinking about EOLC, facilitate communication, facilitate decision‐making	About Palliative Care (Podcast, webpage and video) What, how, where, payments, support for family/carer, language support	Yes 19 community languages: Arabic; Traditional Chinese; Simplified Chinese; Croatian; Dutch; Greek; Hebrew; Hindi; Italian; Karen; Macedonian; Maltese; Polish; Russian; Serbian; Spanish; Turkish; Vietnamese; Yiddish	No	No	No
Govt.	Queensland Health, 2018 [43]	State	1. HCP and services that embed ACP into routine practice 2. Hospital and Health Services (HHS)	Type: print Aim: Facilitate thinking about ACP, facilitate communication, facilitate decision‐making	Advance Care Planning—Clinical Guidelines Guidelines to establish best practice principles for ACP. *Planning process:*	*1. Topics to be covered during ACP discussions should include:* a. feelings, beliefs or values that may be influencing the person's preferences and decisions b. person's needs for religious, spiritual or other personal support c. wishes in relation to funerals, the handling of their body (including cultural needs), and their beliefs or values about organ or tissue donation. *2. Communication Examples (question list): when spiritual/existential concerns are raised* 3. Specific issues to consider when initiating conversations and discussing ACP with CALD community members: effects of migration, cultural aspects, need to approach sensitively, understand where religion fits within spectrum of decision‐making for each group, not to assume anything 4. Each culture has rituals and mechanisms for dealing with death—there are common interests that may serve as starting points for the ACP discussion. 5. In most groups, family has been the main source of security, assisted by adherence to religious or spiritual beliefs.		Yes	No
					1. Identifying beneficiaries 2. Assessing needs and capacity 3. Discussing People involved Setting and location Communication skills for introducing and discussing ACP Preparing for discussion Topics to be covered 4. Planning 5. Coordinating 6. Reviewing *Communication Examples* (list of questions) for				
					1. Unwell but stable patient 2. Introducing ACP				
Govt.	Queensland Health, 2022 [44]	State	HC staff: generalists and novice clinicians	Type: print Aim: facilitate thinking about EoL, facilitate communication, facilitate decision‐making	Care Plan for the Dying Person: Health Professional Guidelines Best Practice Multidisciplinary care at end of life: 1. Commencement and authorisation of Care Plan for Dying Person (CPDP) 2. Initial assessment 3. Ongoing assessment 4. Care after death 5. Risk management considerations	No	*1. Use of cultural guidance or interpreter to overcome language or cultural differences* *People who may usually speak and understand English at home, may revert to their language of origin at time of illness or stress* *2. Initial assessment: S*pecific information needs, communication styles/manners and cultural/spiritual beliefs of the person and family/carers to be considered ‐ Discussion topics to include a person's spiritual, cultural, social, emotional and practical needs now, at the time of death and after death. ‐ Ask for list of customs/values that are important in relation to dying ‐ Ensure availability of interpreter if needed ‐ Providing written information about services/treatment in the person's preferred language where possible. *‐ Spiritually and culturally appropriate support:* referral to a spiritual carer, chaplain or cultural advisor with their consent. Referral to Social Work may be considered. *3. One of **Ten key elements for best care of the dying*** **:** The person and relative or carer or advocate should have the opportunity to discuss their wishes, feelings, faith, beliefs and values	Yes	No
Govt.	ACT Health, 2022 [45]	State/Territory	Consumers	Type: Electronic media (Website), Aim: facilitate thinking about EOLC, facilitate communication, facilitate decision‐making	End of life and palliative care: a. What is EOLC, what is Palliative care/When/Who b. Where can I have palliative care? (Services available in various settings) c. Making choices—ACP/Organ and Tissue Donation/Voluntary Assisted Dying (illegal in ACT) d. EOLC for specific groups—First Nations, LGBTA+, incarcerated, disabled and demented, parents who lose a child Using the SPICT Tool as a trigger for resuscitation planning (health staff) SPICT is a guide describing clinical signs that can help identify patients who are at risk of deteriorating and dying from one or more advanced conditions (www.spict.org.uk).	No	Spiritual care (for consumers) ACT Palliative Care can connect consumers to different faith communities in all healthcare settings including home. Many public hospitals have access to a spiritual support team Most aged care facilities have access to spiritual support	No	No
Govt.	SA Health, 2016 [46]	State	Consumers	Type: electronic (webpage) Aim: facilitate thinking about EOL, facilitate decision‐making	Create my Advance Care Directive ‐ Ways of completing ACD: By hand, electronically, then print, Online ‐ Information about ACD DIY Kit ‐ Appointing Substitute Decision Makers	Yes 90 community languages: Afrikaans; Albanian; Amharic; Arabic; Armenian; Assamese; Azerbaijani; Basque; Bengali; Bosnian; Bulgarian; Simplified Chinese; Traditional Chinese; Croatian; Czech; Danish; Dhivehi; Dutch; Estonian; Finnish; French‐Canadian; French—Europe; Galician; Georgian; German; Greek; Gujarati; Haitian Creole; Hausa; Hindi; Hungarian; Icelandic; Igbo; Indonesian; Irish (Gaelic); Italian; Japanese; Kannada; Kazakh; Khmer; Korean; Kurdish; Kyrgyz; Lao; Latvian; Lingala; Lithuanian; Macedonian; Malagasy; Malay; Malayalam; Maltese; Māori; Marathi; Mongolian; Myanmar (Burmese); Nepali; Norwegian; Pashto; Persian (Farsi); Polish; Portuguese; Punjabi; Romanian; Russian; Samoan; Sepedi; Serbian; Sesotho; Shona; Slovak; Slovenian; Somali; Spanish—Europe; Spanish—US; Swahili; Swedish; Tamil; Telugu; Thai; Tigrinya; Turkish; Ukrainian; Urdu; Vietnamese; Welsh; Xhosa; Yaruba; Zulu	No	No	No
Govt.	WA Health, 2022 [47, 48, 49, 50]	State	Consumers (CALD‐related documents); Healthcare staff	Type: electronic media (website) with links to printed media and Videos Aim: facilitate thinking about EOLC, facilitate communication, facilitate decision‐making	‘Goals of Care Form: Discussion tips’ (healthcare professionals) *A. Discussion tips for EOL Planning: (provides a list of questions)* **ASK—TELL—ASK** Model *B. Addressing emotions with empathic responses: **NURSE** Model* **N**ame it, **U**nderstand the message, **R**espect/reassure at the right time, **S**upporting, **E**xploring *C. The **SPIKES** model when breaking bad news*. **S**—SETTING UP the interview **P**—assessing the patient's PERCEPTION **I**—obtaining the patient's INVITATION. **K**—giving KNOWLEDGE and information to the patient **E**—addressing the patient's EMOTIONS with empathic responses **S**—STRATEGY and SUMMARY *D. Things you should say/should NOT say* Guide to ACP (consumers) To prepare for and understand what to consider as consumers complete ACD	1. ACP guide Workbook [41]. (consumers) Activities to help consumers gather thoughts, get started and guides them through ACP process: *Think > Talk > Write > Share* a*. My future care*: What is ACP, importance, what is involved, Activity: Let's get started—your situation *b. Think:* What matters most to me now and if I become less well in future? Activity: Values, beliefs and preferences *c. Talk:* Who can you talk to about ACP? What are some things to talk about? Activity: people to talk to *d. Write:* Who will make treatment decisions for me if I cannot make/communicate my own decisions? ACP‐related documents, Activity: Choosing an ACP document e. Share: Where should I store my ACP documents? Who should I share them with? Activity: Sharing ACP documents—English version provides contact details for interpreter services if required Yes 16 community languages: Afrikaans; Arabic; Simplified Chinese; Croatian; German; Greek; Hindi; Bahasa; Italian; Macedonian; Polish; Punjabi; Spanish; Tagalog; Vietnamese 2. ACP Factsheet [42] (consumers) ‐ What is ACP ‐ Importance ‐ Process: Think > Talk > Write > Share (can go back and forth with these steps) Yes 16 community languages: Afrikaans; Arabic; Simplified Chinese; Croatian; German; Greek; Hindi; Bahasa; Italian; Macedonian; Polish; Punjabi; Spanish; Tagalog; Vietnamese 3. Values and Preferences Form [43] (consumers) A non‐legal form used by consumers to make a record of values, preferences and wishes about future health and personal care, before they are ready for ACP/ACD. Yes 16 community languages: Afrikaans; Arabic; Simplified Chinese; Croatian; German; Greek; Hindi; Bahasa; Italian; Macedonian; Polish; Punjabi; Spanish; Tagalog; Vietnamese 4. Easy Read version of ACP Guide workbook [44] (consumers, English only) ‐ Provides contact details of Interpreter services if required. No Easy‐to‐read version	1. No 2. No 3. No 4. No	1. Yes 2. No 3. No 4. No	1. Yes Consumer group provided content expertise and guidance to shape the document 2. No 3. No 4. Yes Consumer group provided content expertise and guidance to shape the document
Govt.	WA Health [51]	State	Consumers	Type: electronic (website) with videos and podcasts Aim: facilitate thinking about EOLC, facilitate communication, facilitate decision‐making	‘Have The conversation’ (Video) ACD Planning: gathering/discussing thoughts with doctors and loved ones.	Yes One community language Hindi	No	No	No
Non‐govt.	Multicultural Communities Council of Illawarra (MCCI), 2022 [52]	State/territory and local	Consumers	Type: print (booklet) Aim: Facilitate thinking about EOLC, facilitate communication, facilitate decision‐making	What is ACD and process: Think > Talk > Record	Yes 22 community languages: Arabic; Burmese; Chinese; Croatian; Dari; Filipino; Greek; Hindi; Italian; Karenni; Korean; Macedonian; Maltese; Polish; Portuguese; Punjabi; Russian; Serbian; Spanish; Turkish; Ukrainian; Vietnamese	No	No	No
Govt.	SWS PHN, 2023 [53]	Local	Consumers	Type: electronic (webpage) Aim: facilitate communication, facilitate decision‐making	Advance Care Planning ‐ What is it? what will my GP do now? what can I do? ‐ Legal definitions of ACP, ACD, palliative care plan, Enduring Guardian/Person Responsible ‐ Questions to ask my doctor: What should my plan include? Who needs to see my plan? How long does my plan need to be? Will you give me advice about my plan? 5 Where do I keep my plan What if I want to change my plan? How do I know if my plan is ‘finished’? What happens if my plan changes without my knowledge. ‐What supports are available? ‐Where can I learn more?	Yes Three community languages: Arabic; Chinese; Vietnamese	No	Yes	Yes Endorsed by ‘Community Advisory Committee’
Govt.	Carer Help, 2022 [54]	Unspecified	Carers	Type: print Aim: facilitate thinking about EOLC, facilitate communication, facilitate decision‐making	Carer Info Pack 1—Getting Started	Yes Nine community languages: Arabic; Greek; Hindi; Italian; Punjabi; Simplified Chinese; Traditional Chinese; Spanish; Vietnamese	No	No	Yes Reviewed by a peak body of voluntary carers
					1. *List of questions based on common concerns that carers of people with advanced disease have (conversation starter with healthcare staff)* a. Questions about where to provide care b. Questions about who to call: c. Questions about your own well‐being 2. *Managing Communications* **Communication tips**				
					Be clear about what you want to say. Helpful to make some notes beforehand. Listening is a very important part of good communication. Ask clarifying questions. If the topic is very difficult for you, ask if you can think about it—make sure you return to the conversation. You have a right to share your feelings. Be honest about how you feel and what you need. Might need to start the conversation several times. When making a point try explaining the effect something has on you rather than blaming the other person. Don't be afraid to ask for specific types of help.				
					**Important conversations** **Communicating with doctors and nurses**				
Non‐govt.	CARE Search ‐PCKN [55]	Unspecified	Consumers	Type: Electronic media (Website) with links to print media, videos and podcasts Aim: Facilitate thinking about EOLC, facilitate communication, facilitate decision‐making	XX	Multimedia: videos and podcasts (community languages) to provide an overview of palliative care. (consumers) **Videos** talk about palliative care and the changes and situations that consumers may experience including: ‐How to care ‐Groups with specific needs ‐At the end ‐Bereavement, Grief and Loss. **Podcasts** cover specific issues including: ‐ What is palliative care? ‐ Planning ‐ Types of services available ‐ Pain and symptom management ‐ What matters most ‐ Financial services. Yes Eight community languages: Arabic; Cantonese; Croatian; Greek; Italian; Mandarin; Spanish; Ukrainian	No	No	No
Non‐govt.	End‐of‐life Essentials, 2023 [56, 57]	Unspecified	Healthcare staff	Type: Print Aim: Facilitate thinking about EOLC, facilitate communication, facilitate decision‐making	2. Planning End‐of‐Life Care—Goals of Care Checklist to make sure Goals of Care are established and delivered.	No	1. Checklist: EOLC for diverse communities [50]: ‐ Self‐reflection on openness to diversity ‐ Appropriate use of the interpreter ‐ Faith/beliefs/ceremonies/involving religious representatives ‐ Patient‐centred behaviour ‐ Resources provided: National Palliative Care programmes relevant to diversity 2. Planning End‐of‐Life Care—Goals of Care (51) ‐ Consider learning more about cultural competence ‐ Find out more about cultural practices/beliefs about illness and death in relation to an ethnic/religious group encountered in professional practice (All groups are not the same). If in doubt, always ask. ‐ Patient‐centred behaviour and appropriate use of an interpreter ‐ Ask a patient if they would like to see a priest, minister, imam, rabbi or other. ‐ Discuss how their spiritual beliefs might impact upon their care preferences or what happens to them once they die. ‐ Remember the patient may not adhere to all aspects of their reported faith—ask what is personally meaningful to them. ‐ Support the wishes of patients, families and carers who want to include religious or cultural practices into their care. This may include ceremonies, singing or foods. Don't be afraid to ask one of those present the significance of the ritual. ‐ ‘Family’ is who the patient says it is. Notions of kinship vary depending on cultural background.	1. No 2. No	1. No 2. No
Non‐govt.	Palliative Care, Australia [58, 59]	Unspecified	1. Consumers 2. Consumers and healthcare staff	Type: Print Aim: facilitate thinking about EOLC, facilitate communication, facilitate decision‐making	1. Factsheet ‘How palliative care can help’ [52] 2. ‘What matters most for older Australians’ (Discussion Starter Series and card‐pack) [53]	1. Yes 20 community languages: Amharic; Arabic; Chinese; Farsi; Greek; Hindi; Italian; Karen; Polish; Portuguese; Punjabi; Russian; Serbian; Somali; Spanish; Tagalog; Tigrinya; Turkish; Urdu; Vietnamese 2. Yes 11 community languages: Arabic; Croatian; Greek; Hindi; Italian; Maltese; Polish; Simplified Chinese; Spanish; Ukrainian; Vietnamese	No	1. No 2. Yes	1. No 2. Yes Pilot study (focus group and interviews) with consumers post‐preparation

Twenty‐one resources targeted consumers, eight targeted healthcare staff and one both consumers and healthcare staff. The most represented media type was print media (*n* = 22).

Fifteen resources were available in languages other than English, covering a total of 91 community languages (Supplementary File 4) [19, 21, 22, 42, 46, 47, 48, 49, 51, 52, 53, 54, 55, 58, 59]. Five resources were available in easy‐to‐read versions in English [37, 38, 39, 40, 50]. These resources had specifically mentioned that they were designed to be easy‐to‐read. Only eight documents referenced their evidence base [20, 23, 35, 43, 44, 47, 53, 59]. Consumer involvement was evident in the development of 12 of the resources (see Table 2) [21, 35, 36, 37, 38, 39, 40, 47, 50, 53, 54, 59]. Eleven of these resources were targeted towards consumers [21, 36, 37, 38, 39, 40, 47, 50, 53, 54, 59] and one was targeted towards [35] healthcare staff.

### 3.2 Approaches Recommended to Support Engagement of CALD Communities With ACP

Out of 30 documents available, only 10 documents provided recommendations for engagement approaches with CALD community and were subject to further analysis. Inductive thematic analysis identified 11 sub‐themes across the included documents, which were subsequently mapped into four themes (Table 3).

i. *Theme 1: Practising culturally sensitive and person‐centred approach*

**Table 3:** Sub‐theme considerations across 10 documents.

Title and year	Sub‐theme considerations across 10 documents with recommendations for CALD community										
	Appropriate use of interpreters	Person ‐centred care	Provide information in accessible format	Develop cultural awareness and competence of healthcare staff	Provide multidisciplinary support for effective communication	Provide opportunity for active engagement to CALD community	Establish preferred decision‐making approach	Consider literacy and health literacy	Employ bilingual/cultural staff and diversity champions	Co‐produce and co‐design with CALD community	Responsive healthcare provision
End of Life Care and Decision‐making, 2021 [35]	×	×	×				×		×		×
Advance Care Planning—Clinical Guidelines, 2018 [43]		×					×				×
Care Plan for the Dying Person—Health Professional Guidelines, 2019 [44]	×	×	×		×	×			×		
Asking Questions Can Help, 2016 [36]	×	×									
Cultural, Spiritual or Other Needs, 2022 [45]		×									
Supporting Inclusive EOLC: Older People from CALD Backgrounds, 2022 [23]	×	×	×	×		×		×	×	×	×
Delivering and Supporting Comprehensive EOLC: a user guide, 2021 [20]		×			×	×					
Checklist: End‐of‐Life Care for Diverse Communities, 2023 [56]	×	×	×		×	×	×				
Checklist: Planning End‐of‐Life Care‐Goals of Care, 2023 [57]	×	×		×			×				
Death, dying and palliative care, 2020 [41]		×	×			×					

Practising culturally sensitive and person‐centred approach to communicate with CALD consumers was a major theme highlighted across all 10 resources [20, 23, 35, 36, 41, 43, 44, 45, 56, 57]. This theme comprised of recommendations that were grouped into five sub‐themes: (a) practising person‐centred care [20, 23, 35, 36, 43, 44, 45, 56, 57]; (b) providing opportunities for active engagement of CALD consumers [20, 23, 41, 44, 56]; (c) establishing preferred decision‐making approach [35, 43, 56, 57]; (d) responsive healthcare provision [23, 35, 43] and (e) co‐designing and co‐producing resources, services and programmes with CALD community [20, 23].

*Sub‐theme a: Practising person‐centred care*

Within the 10 resources, various approaches were recommended for practising person‐centred care. Healthcare providers were advised not to make any assumptions about people from CALD backgrounds [43] and to engage in self‐reflection on their openness to diversity [56]. ACP discussions were recommended to include religious, cultural, emotional and practical needs [20, 35, 36, 41, 43, 44, 45, 56, 57], while being mindful that CALD individuals may not adhere to all aspects of their reported faith and all ethnic/religious groups are not same [57]. Providers were also encouraged to explore cultural practices and beliefs surrounding illness and death specific to the ethnic/religious group encountered in professional practice [57] and to consider the possible effects of migration on health beliefs and decision‐making [43]; mandatory staff introduction and inquiry on preferences for pronouns for consumers and families from CALD backgrounds [56]; and showing compassion, sensitivity and empathy [43, 56].

*Sub‐theme b: Providing opportunities for active engagement of CALD consumers*

Providing opportunities to consumers from CALD backgrounds to actively participate in ACP [20, 23, 41, 44, 56] was encouraged largely by asking open‐ended and inquiring questions such as ‘what matters to you?’ [20] or ‘what is important to you?’/‘what is the most important thing I should know about you?’ [44]. Another recommendation for healthcare staff was to read ACP‐related information with CALD consumers in accessible formats, encouraging them to ask questions and fostering a two‐way conversation [41].

*Sub‐theme c: Establishing preferred decision‐making approach*

Recommended strategies for establishing a decision‐making approach (autonomous vs. collective, any elected substitute decision‐maker, or role of culture/religion) [35, 43, 56, 57] emphasised the importance of understanding cultural nuances and individual preferences. This included recognising where religion fits within the spectrum of decision‐making for each cultural group [43, 57] and exploring whether the approach is autonomous, collective or delegated, with the assistance of interpreters or trained cultural health workers [35]. Healthcare providers were encouraged to confirm whether patients had nominated someone to make decisions on their behalf if they became unable to do so or if they had discussed their wishes with that person [56]. Additionally, mapping the patient's close ties and family was suggested as a starting point for identifying potential alternative decision‐makers and determining who should be involved in discussions about the patient's care [57]. These strategies aim to ensure that decision‐making processes are culturally sensitive, inclusive and tailored to individual needs.

*Sub‐theme d: Responsive healthcare provision*

Responsive healthcare provision was considered imperative to support culturally safe, inclusive and respectful ACP engagement [23, 35, 43]. For example, a proactive and flexible aged care system that responds to the needs of existing and emerging diverse groups [23]. Resources suggested that aged‐care providers should engage with the local community and stakeholders to identify emerging needs and how service delivery models can be adapted to respond to those needs in an inclusive approach to care. Suggested strategies included engaging with carers from CALD backgrounds in the community to share their lived experiences of palliative care with staff, highlighting what worked well for them, including: bereavement care and support; recognition of cultural beliefs; and participation in end‐of‐life decision‐making. Further strategies were to encourage staff to seek feedback from older people from CALD backgrounds in a culturally inclusive way that acknowledges language, literacy and cultural considerations. Finally, the importance of recognising the influence of ethnicity, religion, sexual orientation, gender, migration status, socio‐economic factors, disability and age on an individual's approach to engaging in ACP was noted. Of particular focus was addressing any influence of traumatic events through trauma‐informed care (wars, other pre‐migration experiences, financial hardship and torture) [23].

*Sub‐theme e: Co‐designing and co‐producing resources, services and programmes with CALD communities*

Co‐designing and co‐producing with the CALD community was identified in one resource as important for services to effectively meet the specific needs of older people with diverse characteristics and life experiences, their families, carers and representatives in a respectful and inclusive way [23]. This resource described of the need to consult, partner or collaborate with advocacy agencies, community groups or organisations to plan and deliver culturally sensitive and inclusive care at the end of life. Approaches to co‐design proposed included establishing an ACP working group with CALD members, demographic assessment of the needs of the community, and partnering with local services to co‐design and plan end‐of‐life care to meet emerging population needs and promoting a culture of inclusivity across health service organisations [23].

*ii. Theme 2: Supporting communication needs*

Supporting communication needs of CALD consumers was identified as a major theme spanning seven resources [23, 35, 36, 41, 44, 56, 57]. Recommendations were grouped into three sub‐themes: (a) practising appropriate use of interpreters [23, 35, 36, 44, 56, 57], (b) provision of information in accessible formats for the CALD community [23, 35, 41, 44, 56] and (c) considering the literacy and health literacy level of individual consumers [23].

Recommended approaches to promote the use of interpreters included booking interpreters beforehand for planned ACP conversations [56], avoiding informal use of untrained interpreters while communicating about clinical information [35] and, where culturally appropriate, maintaining eye contact with the patient while listening to interpreter [56]. To provide information in accessible formats, suggested approaches [23, 35, 41, 44, 56] emphasised considering patient‐specific information needs and communication styles [44], ensuring information is communicated in a language the patient understands and in appropriate formats including online, via hardcopy, or verbally [23]. Other recommendations included utilising simplified versions with visual aids and picture formats [35] and avoiding medical jargon or overly complex language [41].

*iii. Theme 3: Staff recruitment and training*

Staff recruitment and training was discussed in five resources [23, 35, 44, 56, 57] in relation to two sub‐themes: (a) developing cultural awareness and competence of healthcare staff [23, 56, 57] and (b) employing bilingual/cultural staff and diversity champions for specific purposes [23, 35, 44]. Developing cultural awareness and competence of staff was recommended to explore the needs and preferences of consumers from CALD backgrounds, particularly in relation to recognising end of life and documenting ACP discussions [23, 56, 57]. Employment of bilingual/cultural staff and diversity champions was recommended to overcome language or cultural differences during ACP discussions [35, 44] and providing culturally and linguistically appropriate care at the end of life [23].

*iv. Theme 4: Multidisciplinary support for effective ACP communication*

The importance of investigating and providing collaborative, multidisciplinary support tailored to a patient's specific needs to address mental, psychological, spiritual and cultural health was identified as a key issue in three resources [20, 44, 56]. Multidisciplinary support included medical specialists such as mental health professionals, allied health professionals including psychologists, social workers, spiritual care practitioners, speech pathologists and occupational therapists [20, 44, 56]. Having access to a multidisciplinary team that helped in effectively communicating patient needs and concerns was essential to safe and high‐quality care at the end of life. It was acknowledged that people from diverse backgrounds may choose to discuss goals with people who provide different types of support, for example, spiritual care practitioners/cultural advisors/chaplains who can help other members of the healthcare team understand the patient's spiritual needs for consideration during care planning [20]. Healthcare staff were asked to consider any benefit from assessment by mental health staff while engaging with CALD consumers at the end of life, but no further details were provided [56]. Consideration of legal support to assist with EOL planning was mentioned with no further exploration [56].

## 4 Discussion

### 4.1 Key Findings

Our study highlighted that despite a high volume of documents, few have been co‐designed with communities, or provided specific recommendations for the CALD population and most targeted healthcare staff. Among the documents that did address CALD communities, none provided targeted information about the practices or needs of any specific community. There was a notable lack of documents directed at health consumers. Few documents identified to have consumer involvement in their preparation and targeted two‐way communication between consumers and healthcare staff.

Our findings align with wider evidence of the gaps in support for both health professionals and consumers from CALD backgrounds to engage in ACP. An umbrella review of systematic reviews reported lack of knowledge and skills among healthcare staff as a major barrier to ACP [60]. Nurses felt ill‐prepared and identified a lack of formal education to carry out conversations about care at the end of life with elderly immigrants in Australia, relying on tacit learning from experienced colleagues [61]. In our recent research examining cancer care in Australia, healthcare staff reported a lack of ACP communication resources to provide to consumers from CALD backgrounds. They reported that resources were not available in all languages and of those available, staff had no knowledge of how to access them [62]. They were also not aware of resources available for healthcare care staff to prepare them for ACP communication with people with cancer from CALD backgrounds. Further work has established that consumers from CALD backgrounds with a cancer diagnosis were not aware of resources to support them to engage in ACP [63]. This was in stark contrast to the identification of availability and easy access of numerous community language documents targeting CALD consumers from websites in this document analysis. Similarly, documents targeting healthcare staff were easily accessible from websites.

Beyond our findings of the availability of resources to promote ACP uptake in CALD communities, evidence of the extent of uptake and perceived value of the resources is required. The availability and easy accessibility of 30 publicly available resources to promote ACP engagement in this study suggests that work is required to ensure health providers and consumers are aware of these resources. Greater awareness and adoption may be achieved by emphasis on the implementation of available resources at service level and increasing awareness of healthcare staff around their location and access. Additionally, establishment of a centralised repository of resources within state and federal health systems may facilitate access to up‐to‐date information.

Approaches to engage CALD communities in ACP were predominantly derived from resources targeting healthcare staff. Consumer facing documents were direct translations of ACP guides, which fail to consider the range of cultural, religious or spiritual beliefs affecting choices for care at the end of life [64, 65]. Internationally identified approaches to engage CALD communities with ACP exhibit usage of standalone culturally tailored documents [66], in combination with facilitation [67, 68] in combination with translated/easy‐to‐read documents [69]. Each of these approaches has been shown to increase engagement with ACP [66, 67, 68, 69]. Given that Australia lacks availability of CALD‐tailored documents targeted at consumers at this stage, it is recommended that their development should be a priority.

Consumers have previously identified barriers to uptake of ACP as fear of discussing their relative's end of life, lack of ability to engage in ACP, and not knowing who was responsible for initiating conversations about ACP [60]. Few documents that were identified to have consumer involvement in preparation phase targeting two‐way communication in our study may be trialled for efficacy in engagement with ACP [21, 35, 36, 37, 38, 39, 40, 47, 50, 53, 54, 59]. Involving consumers in preparing resources may facilitate production of documents targeted to the needs of CALD communities. A recent Australian study with Chinese‐speaking community members who participated in a co‐designed Chinese ACP educational workshop identified awareness of ACP process being related to its uptake, albeit a modest uptake of ACP in a small follow‐up sample [70]. Co‐design methodology was successful in exploring perceived needs, barriers to ACP uptake, knowledge gaps and perceived changes required to current practice. Chinese language tools were created to address identified cultural barriers (poor knowledge of ACP, difficulty understanding ACP documentation and use of culturally insensitive terms). Similar co‐design approaches may be adopted with other community groups in Australia. Evaluation of developed resources in terms of facilitating CALD consumer engagement around ACP is recommended.

Prior research has highlighted that efforts to promote skills of healthcare staff in having ACP conversations with CALD communities is a priority [60, 61]. Documents in this study recommend strategies at various levels targeting consumers, healthcare staff, organisation and/or community separately or in combination to engage CALD community. International research suggests that multilevel strategies may be useful. For example, a combination of provider prompts (physician education and desk book) and organisational prompts (checklist on chart, preprinted reminder stickers to reintroduce issue during visit and enhanced availability of ACDs) [71]; and a multilevel intervention ‘My Life, My Way’ to increase engagement of diverse population in an urban community hospital in the United States targeted consumers, healthcare staff (clinical, allied and support staff), organisation and community resulting in improved engagement with ACP [72]. This strategy is utilised in person staff trainings, CALD tailored documents and multicultural staff. Future work/research in Australia should build on this international work to address the gaps in documents our work has highlighted. Additionally, there is a need for a review of sector regulatory standards (Palliative Care Standards 1 and 7), which may create opportunities for legal accountability at the healthcare practitioner level, serving as a potential assurance mechanism for mandatory sector involvement [73].

### 4.2 Limitations

This document analysis is limited by its time frame that it extends to June 2023 due to the extensive process of searching, extracting and synthesising resources from disparate sources. When scoping more recent resources published between July 2023 and December 2024, we noted the additional presence of (a) recommendations for consideration of intersectionality rather than only cultural/ethnic diversity [74], (b) availability of interactive software on website to determine specific and total CALD community demographics in area of practice for healthcare staff (to determine specific needs) [75], and (c) self‐reflection forms for clinicians and nurses about culturally sensitive communication with CALD community with regard to palliative care and ACP [76].

We are limited by the search methods used where we used a wider search term and took an expansive search across a range of organisations. It is possible that various communities have developed bespoke documents that are not easily and/or publicly available and so have been missed in this analysis. In addition, our document analysis involves an assumption that CALD communities find the document/media types we included here (print, websites, podcasts and videos) useful. It is possible that there are some CALD communities where sharing information orally by face‐to‐face encounters is relevant/culturally normative, and/or people prefer to seek information from community members/elders rather than a resource by a spokesperson or body they are unfamiliar with.

## 5 Conclusion

Whilst many resources have been developed to facilitate uptake of ACP among the general population, a limited number of resources provide specific recommendations or support for working with CALD communities. Few resources reported the use of co‐design with communities despite recommendations to do so. Targeted information both for consumers and care providers is required to provide culturally relevant and inclusive support that promotes engagement in ACP towards person‐centric care. In addition, examining the implementation of these resources among consumers and clinicians to explore their value, relevance and accessibility is vital.

## Author Contributions

Reema Harrison and iCanCare project team conceived the study. Two reviewers (Ashfaq Chauhan and Maha Iqbal) completed the database search. Eligibility criteria were developed by two reviewers in consultation (Reema Harrison and Ashfaq Chauhan). Two reviewers performed a second search (Upma Chitkara and Ashfaq Chauhan). Preliminary data extraction was done by one reviewer (Mary Li) followed by refinement by second reviewer (Upma Chitkara). One reviewer (Upma Chitkara) completed data analysis and writing of the manuscript. Reema Harrison, Ashfaq Chauhan, Ramya Walson and Ursula Sansom‐Daly provided feedback during analysis and reporting and reviewed the manuscript. Nadine Elkabbout, Vitor Rocha and Misbah Faiz provided feedback on the final draft of the manuscript as co‐authors.

## Ethics Statement

The authors have nothing to report.

## Conflicts of Interest

The authors declare no conflicts of interest.

## Permission to Reproduce Material From Other Sources

Only publicly available data was utilised in this study.

## Supporting information

SupplementaryFiles_1to4_DocAnalysis_ACP.

## Data Availability

Data sharing is not applicable to this article as no new data were created or analysed in this study.
